# Sustainable Collagen Blends with Different Ionic Liquids for Resistive Touch Sensing Applications

**DOI:** 10.1021/acssuschemeng.3c00052

**Published:** 2023-03-29

**Authors:** Mireia Andonegi, Daniela Correia, Nelson Pereira, Manuel Salado, Carlos M. Costa, Senentxu Lanceros-Mendez, Koro de la Caba, Pedro Guerrero

**Affiliations:** † BIOMAT Research Group, University of the Basque Country (UPV/EHU), Escuela de Ingeniería de Gipuzkoa, Plaza de Europa 1, 20018 Donostia-San Sebastián, Spain; ‡ Center of Chemistry, University of Minho, 4710-057 Braga, Portugal; § Physics Centre of Minho and Porto Universities (CF-UM-UP), University of Minho, 4710-057 Braga, Portugal; ∥ BCMaterials, Basque Center for Materials, Applications and Nanostructures, UPV/EHU Science Park, 48940 Leioa, Spain; ⊥ Institute of Science and Innovation for Bio-Sustainability (IB-S), University of Minho, 4710-053 Braga, Portugal; # Laboratory of Physics for Materials and Emergent Technologies, LapMET, University of Minho, 4710-057 Braga, Portugal; ∇ Ikerbasque, Basque Foundation for Science, 48009 Bilbao, Spain; ○ Proteinmat Materials SL, Avenida de Tolosa 72, 20018 Donostia-San Sebastián, Spain

**Keywords:** collagen, blends, ionic liquid, sustainability, resistive sensor

## Abstract

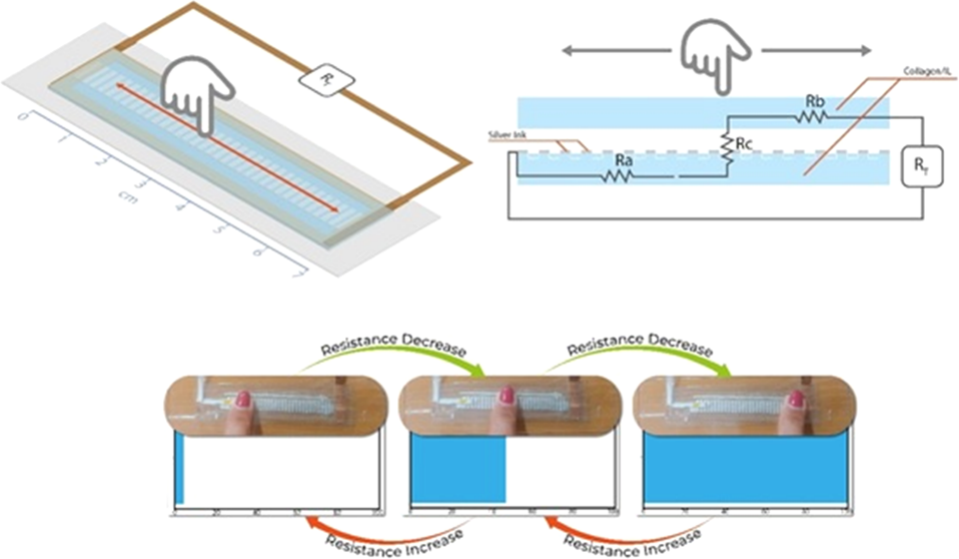

Considering the sustainable development goals to reduce environmental impact, sustainable sensors based on natural polymers are a priority as the large im plementation of these materials is required considering the Internet of Things (IoT) paradigm. In this context, the present work reports on sustainable blends based on collagen and different ionic liquids (ILs), including ([Ch][DHP], [Ch][TSI], [Ch][Seri]) and ([Emim][TFSI]), processed with varying contents and types of ILs in order to tailor the electrical response. Varying IL types and contents leads to different interactions with the collagen polymer matrix and, therefore, to varying mechanical, thermal, and electrical properties. Collagen/[Ch][Seri] samples display the most pronounced decrease of the tensile strength (3.2 ± 0.4 MPa) and an increase of the elongation at break (50.6 ± 1.5%). The best ionic conductivity value of 0.023 mS cm^–1^ has been obtained for the sample with 40 wt % of the IL [Ch][Seri]. The functional response of the collagen–IL films has been demonstrated on a resistive touch sensor whose response depends on the ionic conductivity, being suitable for the next generation of sustainable touch sensing devices.

## Abstract 2

Illustration of the working principle of the sliding sensor based on the resistance variation, with the representation of 3% stages.

## Introduction

Smart and functional materials are of increasing interest for a variety of areas due to their ability to exhibit a functional response variation in a predetermined manner upon changes in their environment.^1−4^ Their technological significance requires the development of advanced functional materials with tailored properties toward specific applications. In this context, in the last years, special attention has been paid to the development of smart and functional materials based on the combination of polymers and ionic liquids (ILs) for a wide variety of applications, ranging from sensors, actuators, energy generation and storage, filtration systems, biomedical, and environmental sensing.^5−7^ The major advantage of IL–polymer-based materials relies on the absence of micro- and nanoparticles to develop a multifunctional composite.^6^

In fact, ILs have gained special attention in the last decade in several fields of knowledge due to their interesting properties.^6,8,9^ ILs, also known as green solvents with melting temperatures below 100 °C, are entirely composed of organic cations and organic/inorganic anions with properties such as negligible vapor pressure, high ionic conductivity, nonflammability, nonvolatility, and a wide electrochemical window (between 4 and 6 V).^10−12^ The applicability of ILs in a wide range of areas is intrinsically related to the simple tunability of the physical–chemical properties by varying cations and anions,^6^ a high number of possible cation and anion combinations existing,^10^ leading to specific functional properties, including magnetic, electrical, and optical properties, among others.^6,13,14^

Thus, ILs have been combined with specific polymers like poly(l-lactic acid) (PLLA),^15,16^ cellulose,^16^ poly(vinylidene fluoride) (PVDF),^17−21^ and poly(methyl methacrylate) (PMMA), leading to a variety of functional characteristics.^22−25^ IL–polymer-based materials have been explored to develop pressure sensing materials based on PMMA/1-ethyl-3-methylimidazolium bis(trifluoromethylsulfonyl)imide ([Emim][TFSI]) with high sensitivity as revealed by a gauge factor of ∼2.73, being stable for >1300 cycles (stable operation)^26^ or [Emim][BF_4_] incorporated in different polymeric matrices including Nafion, PVDF, and thermoplastic polyurethane (TPU), the IL/PVDF composites displaying the best sensing performance (101.20 mV MPa^–1^).^27^ Further, a transparent piezoionic material has been presented based on the thermoplastic elastomer styrene–ethylene–butylene–styrene (SEBS) and the ionic liquid 1-butyl-3-methyl-imidazolium dicyanamide ([Bmim][N(CN)_2_]), the composite resistance varying linearly with the applied force with a pressure sensitivity of approximately 25 kΩ N^–1^ in a dynamic range from 0 to 10 N.^28^

However, besides the great interest in the development of IL–polymer-based materials, most of the studies are based on the combination of ILs with synthetic polymers, there existing a lack of studies regarding their combination with natural polymers,^6,16^ areas in which significant efforts need to be performed to develop sustainable smart and functional materials.^6^

Concern for the environment has increased a special interest in natural and renewable materials in terms of sustainability.^29,30^ In this view, the transition to a circular economy provides an opportunity to establish a more sustainable, efficient, and competitive economy,^31,32^ with one of the aims being the valorization of byproducts. Among the different natural polymers, collagen emerges as a suitable approach for the development of IL–polymer based composites due to its natural availability, as it is the most abundant protein in the extracellular matrix of mammals and generally extracted from the bones and skin of cattle, poultry, or pigs.^33^ Alternatively, collagen can also be obtained from the scales, skin, swimming bubbles, and bones of fish.^34−36^ It provides a high mechanical strength to skin, bone, and other tissues.^37^ In terms of chemical properties, collagen backbone is composed of three parallel polypeptide-α chains in the form of cross-linked fibrils with a triple helix structure that leads to the formation of insoluble fibers responsible for the high mechanical strength and integrity of the extracellular matrix of mammalians. The polarity of the collagen chain also provides interesting electrical properties.^38^

Thus, in the present work, different IL types sharing the same cation ([Ch][DHP], [Ch][TSI], [Ch][Seri]) and anion ([Emim][TFSI]) were incorporated into a collagen matrix, aiming at the development of a pressure sensing materials by tuning the electromechanical response. The influence of the IL type on the morphological, physical–chemical, and thermal properties of the composites was evaluated together with their influence on the mechanical and electrical properties. The potential of the developed materials for sensor applications was demonstrated by the development of a functional prototype.

## Materials and Methods

### Materials

**Scheme 1:**
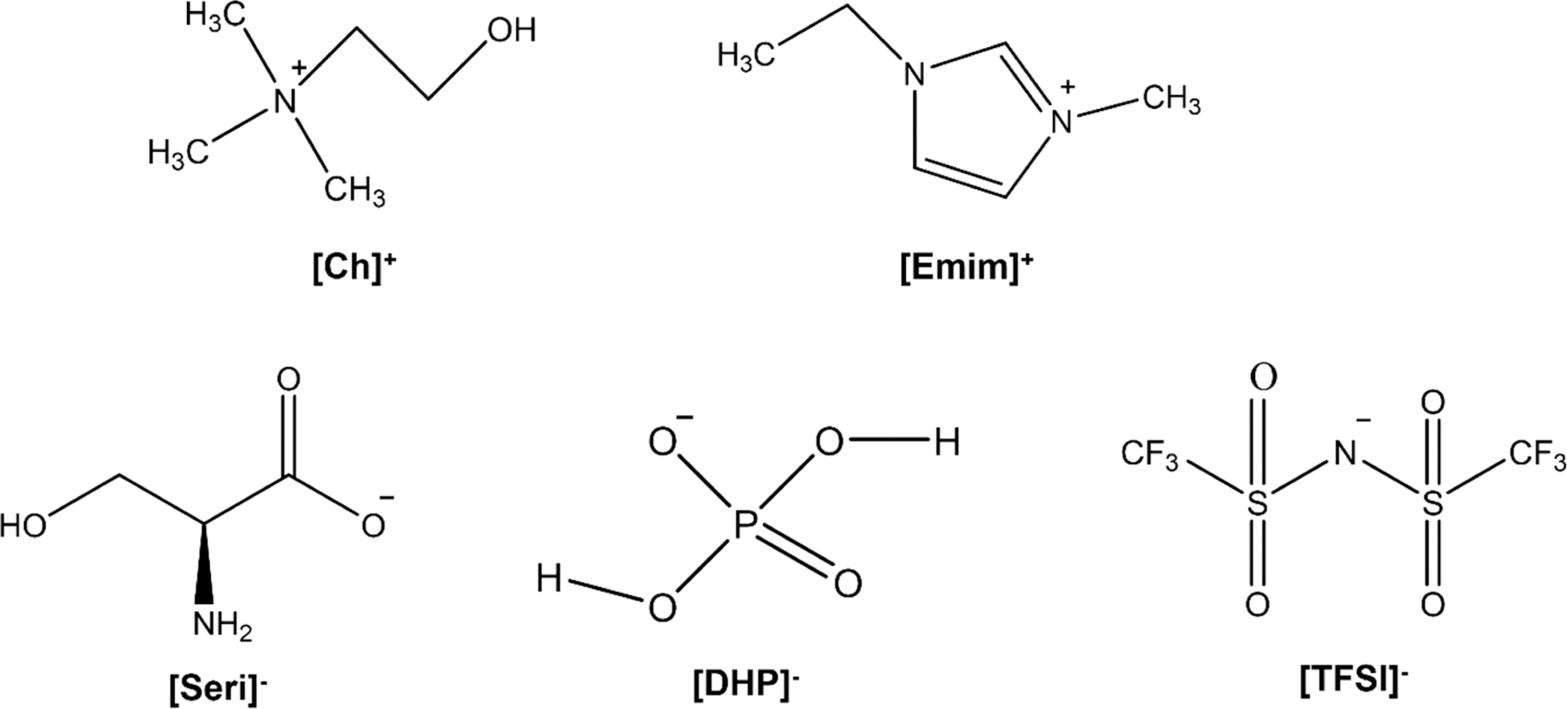
Chemical Structures of the IL Cations and Anions

Collagen was supplied by Proteinmat materials S.L. (Spain). Choline dihydrogen phosphate [Ch][DHP] (>98%), choline derinate [Ch][Seri] (>95, 60% in H_2_O), choline bis(trifluoromethylsulfonyl)imide [Ch][TFSI] (99%), and 1-ethyl-3-methylimidazolium bis(trifluoromethylsulfonyl)imide, [EMIM][TFSI] (99%), were supplied by Ionic Liquids Technologies GmbH (Germany). The chemical structures of the ILs and their main properties are shown in Scheme 1 and Table 1, respectively. Glycerol, pharma grade with a purity of 99.01%, and acetic acid were acquired by Panreac Quimica S.L.U. (Barcelona, Spain).

**Table 1:** Main Properties of the Used ILs

IL	viscosity (cp)	ionic conductivity (mS cm^–1^)	molecular weight	density (g cm^–3^)
**[Ch][Seri]**	11 543.7		208.25	1.19
**[Ch][DHP]**	∼440		201.16	
**[Ch][TFSI]**	49.5 (45 °C)	3.98	368.32	
**[EMIM][TFSI]**	39.4	6.63	391.31	1.52

### Sample Preparation

Collagen blend films with 10, 20, and 40 wt % of [Ch][Seri], 40 wt % of [Ch][DHP], 40 wt % of [Ch][TFSI], and 40 wt % of [EMIM][TFSI] were prepared by solution casting, the composition of the samples being shown in Table 2. Collagen, 20 wt % of glycerol (on collagen + IL basis with the main function of increasing the flexibility of the samples due to the good compatibility with collagen), and the corresponding amount of ILs were incorporated into 0.5 M of acetic acid (1:60 collagen/acetic acid). The mixtures were maintained under stirring at 150 rpm at room temperature for 2 h and then poured into Petri dishes and left to dry at room temperature to obtain the films. Films were designated as 10[Ch][Seri], 20[Ch][Seri], 40[Ch][Seri], 40[Ch][DHP], 40[Ch][TFSI], and 40[EMIM][TFSI], the first number indicating the IL filler content. Films without ILs were considered control samples. All films were conditioned in a climatic chamber, ACS Angelantoni, at 25 °C and 50% relative humidities before testing.

**Table 2:** Relation in Percentage between the Components of IL-Containing Collagen Films

films	collagen (% w/w)	IL (% w/w)	collagen + IL (% w/w)	glycerol (% w/w)
**control**	100	0	80	20
**10[Ch][Seri]**	90	10	80	20
**20[Ch][Seri]**	80	20	80	20
**40[Ch][Seri]**	60	40	80	20
**40[Ch][DHP]**	60	40	80	20
**40[Ch][TFSI]**	60	40	80	20
**40[EMIM][TFSI]**	60	40	80	20

### Sample Characterization

#### Differential Scanning Calorimetry and Thermogravimetric Analysis

Differential scanning calorimetry, DSC, was carried out in a Mettler-Toledo DSC 822. Samples (3.0 ± 0.2 mg) were sealed in aluminum pans to avoid mass loss during the experiment. Filled pans were heated from 25 to 300 °C at a rate of 10 °C min^–1^ under inert atmosphere conditions (10 mL N_2_ min^–1^) to avoid thermo-oxidative reactions.

Thermogravimetric analysis, TGA, was carried out in a TGA/DCS3+ Mettler-Toledo. Dynamic scans from 25 to 800 °C were carried out at a constant rate of 10 °C min^–1^ under a nitrogen atmosphere (10 mL N_2_ min^–1^) to avoid thermo-oxidative reactions.

#### Fourier Transform Infrared Spectroscopy

Fourier transform infrared spectroscopy, FTIR, was performed by using an Alpha II Compact FTIR spectrometer equipped with an attenuated total reflectance (ATR) crystal (ZnSe). A total of 32 scans were carried out at a 4 cm^–1^ resolution.

#### Water Contact Angle

Water contact angle, WCA, measurements of the samples were performed using a DataPhysics OCA 20 contact angle system. A 3 μL droplet of distilled water was placed on the film surface to evaluate its hydrophobic or hydrophilic character. The image of the drop was captured using SCA20 software.

#### X-ray Diffraction

X-ray diffraction, XRD, measurements were performed at 40 kV and 40 mA with a diffraction unit (PANalytical Xpert PRO, Madrid, Spain), generating the radiation from a Cu Kα (λ = 1.5418 Å) source. Data were recorded from 2 to 50°.

#### Scanning Electron Microscopy

Previously to the scanning electron microscopy, SEM, measurements, films were placed on a metal stub and coated with gold using a JEOL fine-coat ion sputter JFC-1100 and argon atmosphere. Samples were observed using a Hitachi S-4800 scanning electron microscope (Hitachi, Madrid, Spain) at a 15 kV accelerating voltage.

#### Mechanical Properties

Bone-shaped samples (4.75 mm × 22.25 mm) were cut, and an Instron 5967 mechanical testing system (Instron, Barcelona, Spain) was used to carry out tensile tests at 1 mm min^–1^, according to the ASTM D 638-03 standard.

#### Electrical Properties

The ionic conductivity value of the collagen blend films was evaluated with a Biologic VMP3 instrument with stainless steel disc electrodes at different temperatures from 25 to 100 °C and a voltage amplitude of 10 mV in the frequency range from 1 MHz to 10 mHz. The ionic conductivity value (σ′) was calculated considering eq 1
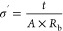
1where *R*_b_ is the bulk resistance obtained from the intercept of the imaginary impedance (minimum value of *Z*″) with the slanted line in the real impedance (*Z*′) and *t* and *A* are the thickness and area of the collagen-based films, respectively.

#### Resistive Touch Sensor Device

**Figure 1:**
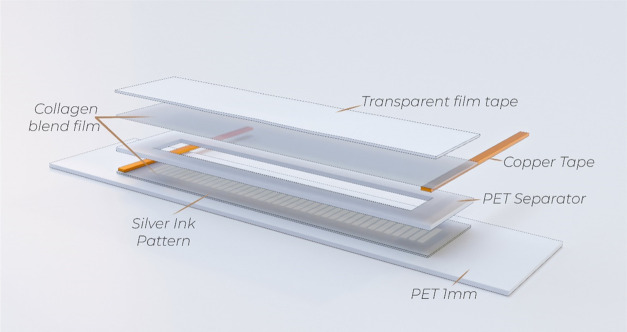
Schematic representation of the assembled sensor.

Figure 1 shows the schematic representation of the resistive touch sensor. Silver electrodes (Novacentrix Metalon HPS-021LV ink) were deposited with the pattern of Figure 1 on the collagen blend films by screen-printing technique, using a manual screen-printing with a mesh of 100 threads by centimeter. The collagen blend films with the deposited ink were cured at 40 °C in an electric convection oven (Pselecta) for 2 h. Then, the films were placed on a poly(ethylene terephthalate) (PET, Goodfellow ES303010/21) substrate of 1 mm of thickness for mechanical stability. On top of the film, a PET separator (Dupont Teijin Melinex 506) of 100 μm of thickness was glued. A second layer of collagen blend film was placed on top of the PET separator with transparent film tape (3M Polyester Film Tape 856). Two strips of copper tape (3M 1181 6 mm) were glued to the top and bottom collagen blend films for electric connection. The pattern enables us to tailor system resistance for an easier readout of the material resistance by the electronic system.

## Results and Discussion

### Physicochemical and Thermal Properties

**Figure 2:**
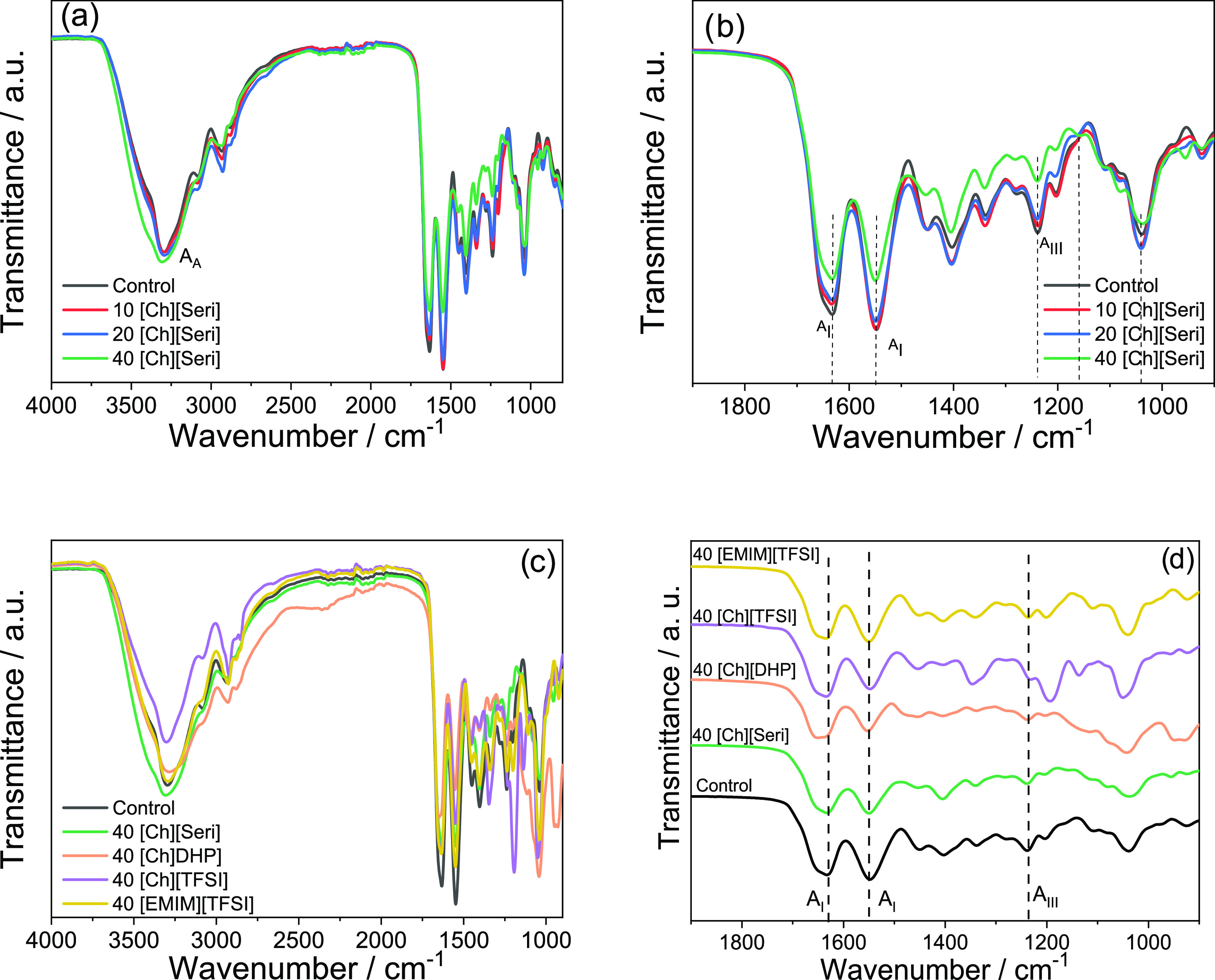
FTIR spectra of collagen films with (a) different contents of [Ch][Seri] from 4000 to 900 cm^–1^, (b) different contents of [Ch][Seri] from 1700 to 900 cm^–1^, (c) 40 wt % of different ILs from 4000 to 900 cm^–1^, and (d) 40 wt % of different ILs from 1700 to 900 cm^–1^.

The interactions among the different components of the films and the effect of the ILs on the integrity of collagen structure were assessed by analysis of the FTIR spectra presented in Figure 2. All of the spectra show the characteristic absorption bands assigned to the peptide bonds in collagen (Figure 2a): 3500–3000 cm^–1^ for amide A (N–H stretching), 1632 cm^–1^ for amide(I) (C=O stretching), 1547 cm^–1^ for amide(II) (N–H bending), and 1238 cm^–1^ for amide(III) (C–N stretching).^39,40^ Furthermore, the amide A band, corresponding to the N–H stretching vibration, appears at 3300 cm^–1^, instead of 3400 cm^–1^, indicating that the NH group is involved in hydrogen bonding.^41,42^ The spectral region between 1200 and 900 cm^–1^ is attributed to the stretching vibrations of C–O bonds in collagen and those related to the hydroxyl groups in glycerol. The band at 1043 cm^–1^ is assigned to the stretching of C–O linkages in C1 and C3 of glycerol, and the band at 1110 cm^–1^ is related to the stretching of C–O in C2 of glycerol.^43^

When choline serinate is added, a decrease in the intensity of the amide(I), amide(II), and amide(III) bands is observed (Figure 2b). These changes confirm the existence of physical interactions, such as hydrogen bonds and ionic interactions, among carboxyl, amino, and hydroxyl groups of collagen, glycerol, and choline serinate, respectively. Moreover, a slight shift in amide(I) (from 1632 to 1630 cm^–1^), amide(II) (from 1547 to 1550 cm^–1^), and amide(III) (from 1238 to 1240 cm^–1^) bands was observed at an increased concentration of [Ch][Seri], indicating stronger interactions between IL and collagen. Furthermore, the addition of choline serinate increases the intensity of the band at 1400 cm^–1^ due to the COO^–^ group of [Seri] anion, and small bands attributed to the ammonium groups of choline are observed at 955 cm^–1^^44,45^ Additionally, shoulders at 1080 and 1160 cm^–1^, corresponding to C–OH groups in serinate and choline,^45,46^ are observed in films with 40 wt % of choline serinate.

On the other hand, the addition of [Ch][DHP], [Ch][TFSI], and [EMIM][TFSI] (Figure 2c,d) also lead to a decrease in the intensity of the amide(I), amide(II), and amide(III) bands, suggesting physical interactions between collagen, ILs, and glycerol. It is worth noting that the relative intensity between amide(I) and amide(II) bands changes when [Ch][DHP] and [Ch][TFSI] are added. In particular, the intensity of the amide(I) band is smaller than that corresponding to the amide(II) band for control films, while the intensity of the amide(I) band becomes greater than that of the amide(II) band for the films with [Ch][DHP] and [Ch][TFSI]. The same trend is observed for the relative intensity between the band at 1450 cm^–1^, attributed to CH_2_ bending vibrations, and the band at 1400 cm^–1^, corresponding to C=O stretching vibrations of those samples.^39,47^ These differences in the relative intensity of FTIR bands suggest that the structure of collagen changes with the addition of [Ch][DHP] and [Ch][TFSI]. Furthermore, the amide(I) and amide(II) bands of 40[Ch][DHP] films are slightly shifted to higher wavenumbers, confirming the strong physical interactions of choline dihydrogen phosphate with collagen and glycerol.^48^ For 40[Ch][DHP] films, a broader band appears at around 950 cm^–1^, related to the ammonium groups (955 cm^–1^) of choline cation^44^ and to the P–OH group (946 cm^–1^) of [DHP] anion.^49^ The shoulder at 1080 cm^–1^ is related to the P=O group of [DHP] anion.^50^ 40[Ch][TFSI] films also present the characteristic band of choline cation as well as those of the triflate anion, such as those related to −SO_2_ at 1342 and 1140 cm^–1^, that related to S–N–S at 1050 cm^–1^, and that related to −CF_3_ at 1192 cm^–1^.^51,52^ 40[EMIM][TFSI] films also show the bands at 1342, 1192, and 1050 cm^–1^ of TFSI.^52,53^ The imidazolium cation bands at 2979 cm^–1^, related to the ethyl chains (CH), and at 3018 cm^–1^, corresponding to the ring (HC–CH and N(CH)N), are overlapped with the wide amide A band (Figure 2c).^54,55^

For a better understanding of the conformational changes in the secondary structure of collagen, the amide(I) profile was analyzed since these interactions can destabilize the collagen native structure according to several reports.^56,57^ Amide(I) contains three major components: a band at 1650 cm^–1^, related to the α-chain/random coil conformation, and two bands corresponding to the β-sheet conformation, which appears at 1615–1630 cm^–1^ and at 1660–1670 cm^–1^.^58^ As shown in Table 3, the α-chain/β-sheet ratio increases with the incorporation of [Ch][Seri] into the formulation up to 20 wt % [Ch][Seri], indicating that choline serinate contributes to the preservation of the native collagen secondary structure and confirming that the protein structure slightly changes as a result of [Ch][Seri]–collagen physical interactions. However, when a higher content of [Ch][Seri] is added, the ratio decreases. Furthermore, the addition of 40 wt % of [Ch][DHP], [Ch][TFSI], and [EMIM][TFSI] also leads to a decrease of the structural native order of collagen, the films being 40[Ch][DHP] and 40[EMIM][TFSI], which show the lowest α-helix/β-sheet ratio values. In any case, α-helix is the main collagen conformation in all hybrid films with ILs, confirming the predominant triple helix structure of collagen.

**Table 3:** Area (%) of Amide(I) Obtained by Fitting, α-Chain/β-Sheet Ratio, and Water Contact Angle (WCA) as a Function of IL Type and Content^a^

samples	β-sheet (%)	α-chain/random coil (%)	α-chain/β-sheet ratio	WCA (degree)
**control**	35.3	64.7	1.8	109 ± 5
**10[Ch][Seri]**	33.3	66.7	2.0	107 ± 3
**20[Ch][Seri]**	33.0	67.1	2.0	99 ± 2
**40[Ch][Seri]**	35.8	64.2	1.8	93 ± 1
**40[Ch][DHP]**	42.6	57.4	1.4	92 ± 2
**40[Ch][TFSI]**	36.5	63.5	1.7	113 ± 2
**40[EMIM][TFSI]**	39.7	60.3	1.5	116 ± 1

a Two means followed by the same letter in the same column are not significantly (*P* > 0.05) different from Tukey’s multiple range test. *N* = 5 was the minimum number of replications.

The film hydrophilic character was analyzed by the measurement of the water contact angle, WCA. As shown in Table 2, WCA values significantly (*P* < 0.05) decrease from 113 to 94° by the incorporation of [Ch][Seri] due to the hygroscopic character of the IL, leading to more hydrophilic surfaces. In the same way, 40[Ch][DHP] films show a similar hydrophilic surface than 40[Ch][Seri]. However, the addition of [Ch][TFSI] and [EMIM][TFSI] leads to hydrophobic surfaces since both are hydrophobic ILs, the most hydrophobic film being 40[EMIM][TFSI].

**Figure 3:**
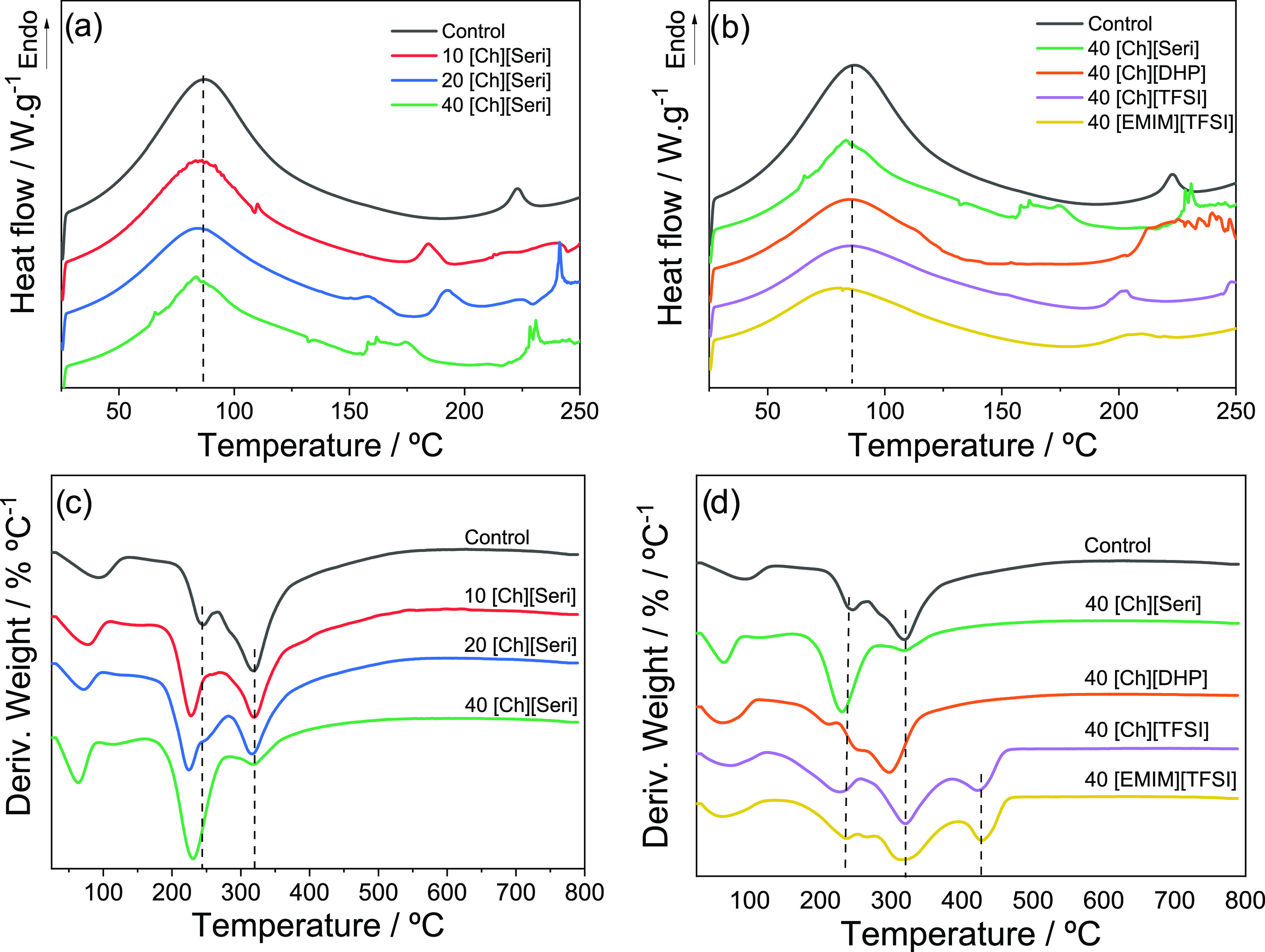
(a) DSC thermograms of collagen films with different contents of [Ch][Seri] and (b) with 40 wt % content of different ILs. (c) DTGA thermograms of collagen films with different contents of [Ch][Seri] and (d) with 40 wt % content of different ILs.

The change in collagen thermal stability caused by the incorporation of the ILs was analyzed by DSC, and the different thermograms are shown in Figure 3.

Characteristic endothermic peaks are found at 85 and 150–250 °C, in accordance with previous studies.^47,59,60^ The first peak is associated with the interfibrillar fraction of water.^61^ In agreement with the physical interactions identified by FTIR analysis, it is worth noting that the values associated with the first peak indicate that collagen fibers remained unchanged, although a slight decrease of the temperature and height of the peak indicates changes in the network hydration.^62^ It can be observed that the denaturation temperature of the samples is lower than that of the control, which may be attributed to the plasticization effect of the IL and its adhesion to the collagen backbone, as also reflected by the mechanical property results. The second peak is related to the transition of the collagen triple helix structure into a random coiled structure by the breakage of intra- and intermolecular hydrogen bonds, which are responsible for the stability of collagen molecules and for the release of loosely bound water. The obtained values are in agreement with the values shown for native fibers.^63^ Differences between the transition temperatures of the samples can be observed, ranging from 175 °C for 40[Ch][Seri] to 225 °C for the control samples (Figure 3a). In the case of [Ch][Seri]-containing samples, the denaturation temperature of collagen decreases by the addition of the IL and thermal events at higher temperature, resulting in narrow or broad endothermic peaks, especially observed in 40[Ch][Seri], which could be attributed to the melting of serine salts, also observed by SEM and XRD analyses.^64^ Additionally, [Ch][TFSI] and [EMIM][TFSI] lead to a decrease in the temperature and the energy input needed to cause the transition (Figure 3b). In the case of collagen with [Ch][DHP], no denaturation peak was observed, probably because it is close to collagen degradation. These differences in the collagen denaturation temperature can be attributed to the strong interaction between collagen and ILs, as observed by FTIR analysis, as well as to the hydrophobic character of [Ch][TFSI] and [EMIM][TFSI].

Regarding thermal stability, decomposition temperatures can change from 200 to 400 °C by varying the anion/cation type, indicating that this property mainly depends on the IL structure and that specific anion and cation play an important role in determining thermal stability. Additionally, it is worth to note that evaporation may occur, so the measured mass loss is a combination of evaporation and degradation. In this work, the influence of the IL amount and anion/cation type on the thermal stability of collagen films was analyzed. Figure 3c shows the thermal degradability of the [Ch][Seri]/collagen systems with an increasing amount of [Ch][Seri]. All samples show three stages of thermal degradation. The first stage of degradation is between 50 and 100 °C, associated with water loss. The second thermal degradation is associated with the evaporation of glycerol (240 °C)^65^ and IL degradation (190 °C).^66^ In the case of the control sample, the evaporation temperature of glycerol is lower than that of pure glycerol, suggesting glycerol–collagen interactions. The addition of [Ch][Seri] shows a decrease in the evaporation temperature up to 220 °C, irrespective of the [Ch][Seri] content, confirming the results obtained by FTIR, where physical interactions between the components were demonstrated. The increase of [Ch][Seri] results in an increase of the evaporation/degradation rate of the samples. The third state of degradation occurs at 320 °C, associated with the thermal degradation of collagen, showing a decrease in the degradation rate with increasing [Ch][Seri].

Concerning the effect of the anion/cation type, Figure 3d shows the thermal degradation of collagen films with 40 wt % of the different ILs. For all samples, the first thermal degradation, between 50 and 100 °C, is associated with water loss. The second phase of thermal evaporation/degradation appears at 220 °C for 40[Ch][Seri], 40[Ch][TFSI], and 40[EMIM][TFSI] systems, while for the 40[Ch][DHP] system, a thermal evaporation/degradation appears at 205 °C, associated with the dehydration of the dihydrogen phosphate anions,^67^ and another transition at 240 °C, associated with the evaporation of glycerol. 40[Ch][Seri], 40[Ch][TFSI], and 40[EMIM][TFSI] systems show a third degradation stage at 320 °C, associated with collagen degradation, while 40[Ch][DHP] system shows this thermal degradation at lower temperatures, 300 °C, leading to a decrease of the thermal stability of collagen. Additionally, the systems with [TFSI] anion, 40[Ch][TFSI], and 40[EMIM][TFSI] show a fourth stage of thermal degradation at 425 °C, related to the decomposition of the IL.^68^

### Morphological and Structural Characteristics

**Figure 4:**
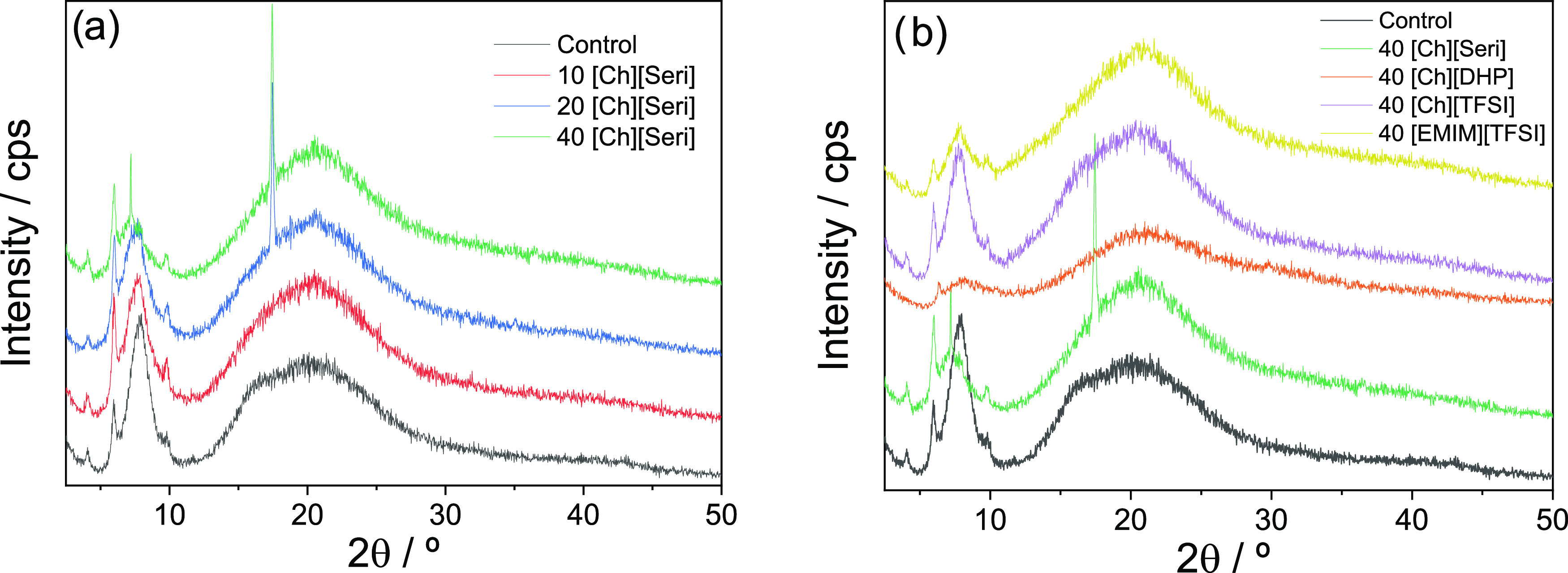
XRD patterns of collagen: (a) with different contents of [Ch][Seri] and (b) with 40 wt % content of different ILs.

Taking into account that ILs can be dissolved and disturb the triple helix structure of collagen during dissolution,^69^ XRD and SEM analyses were carried out to analyze the effect of ILs on the collagen structure. As for XRD analysis (Figure 4), all samples show XRD patterns compatible with nearly amorphous materials, with a broad peak around 2θ = 20°, associated with the diffuse scattering of collagen fibers.

The peak around 2θ = 7° represents the lateral packing distance between collagen chains and is related to the triple helix structure of collagen.^70,71^ It is observed that almost all samples show similar XRD patterns, indicating the prevalence of the collagen structural order. When the [Ch][Seri] content increases, the peak intensity at 2θ = 7° decreases, suggesting the decrease of the structural order in collagen, but no significant differences were observed in the peak at 2θ = 20°. Additionally, a sharp peak appears at around 17° in the films with 20 and 40 wt % [Ch][Seri] (Figure 4a), corresponding to the serine crystals observed on the surface of those films, leading to a heterogeneous system.^64^ In the same way, the addition of [Ch][DHP] decreases the intensity of both peaks, indicating changes in the structural order of collagen, which are in agreement with the changes observed by FTIR and DSC analyses, confirming that [Ch][DHP] decreases the structural order of collagen. In the case of 40[EMIM][TFSI], a slight decrease of the peak at 7° can be observed, indicating a decrease in the structural order of collagen, while the broad band practically does not change. 40[Ch][TFSI] is the film that shows the most similar XRD pattern to that of the control film (Figure 4b).

**Figure 5:**
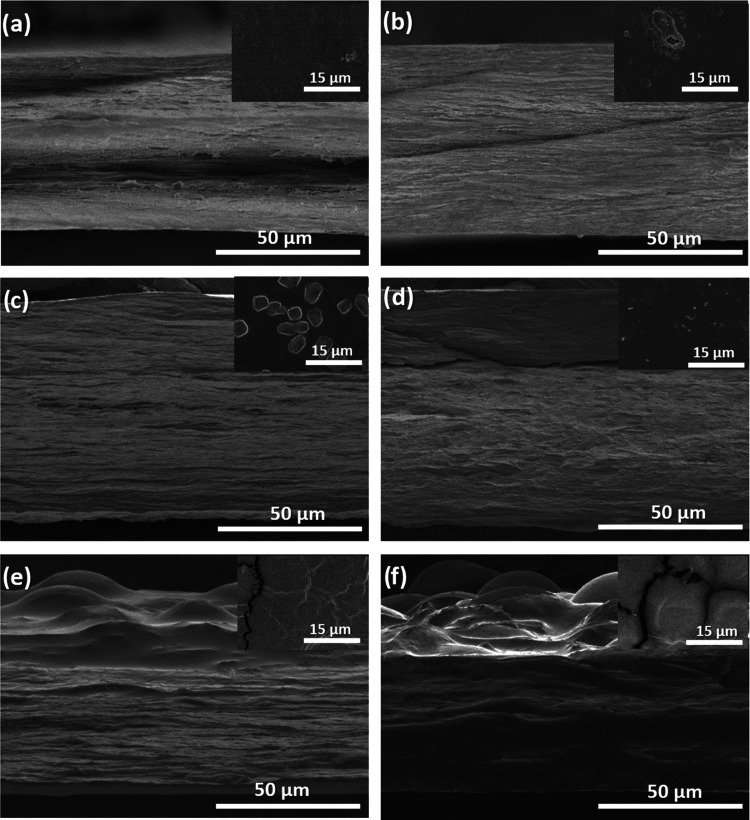
Representative SEM images for surface and cross section of (a) control, (b) 10[Ch][Seri], (c) 40[Ch][Seri], (d) 40[Ch][DHP], (e) 40[Ch][TFSI], and (f) 40[EMIM][TFSI].

Representative SEM micrographs of the surface and cross section of the samples are shown in Figure 5.

The cross-sectional images show that all films are characterized by a compact and homogeneous dense fibrillar structure, even if the images of 40[Ch][TFSI] and 40[EMIM][TFSI] show some differences. In particular, the differences observed in 40[EMIM][TFSI] may be due to the fact that the collagen matrix exudates the excess of IL that covered the surface and does not allow the collagen fibrillar structure to be observed. Additionally, in the cross section of the 40[Ch][DHP] samples, the fibers are not as visible as in the other samples, in accordance with the decrease of the lateral packaging of collagen chains observed by XRD and FTIR analyses. Furthermore, in the surface micrographs of 20 wt % [Ch][Seri]-containing collagen samples, some particles can be observed (Ø, 1 ± 0.5 μm) and their size increases in the 40 wt % [Ch][Seri]-containing films (Ø, 4 ± 2 μm). These results, together with the peak at 17° observed by XRD analysis, indicate that some crystalline structures are formed on the surface of the samples. The surface of 40[Ch][TFSI] and 40[EMIM][TFSI] samples also undergoes changes compared to the surface of the control film. On the one hand, cracks can be observed, which may be due to the hydrophobic character of those ILs, as confirmed by the WCA analysis. On the other hand, the monticules observed in the cross-sectional images may suggest a partial exudation of ILs from the polymeric matrix, which may be due to the immiscibility between the components of the samples.^72,73^ Contrarily, no exudation was observed for the systems containing [Ch][DHP] and [Ch][Seri], confirming the good confinement of these ILs.

### Mechanical Properties

**Figure 6:**
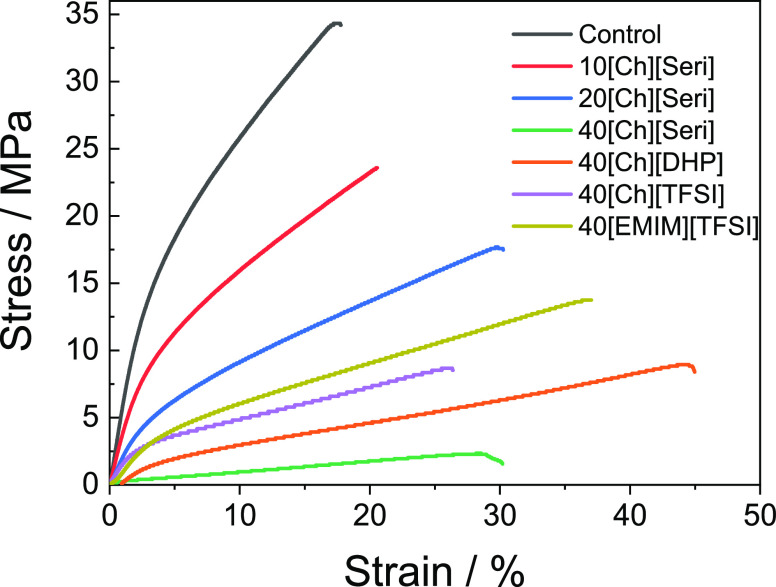
Stress–strain curves of collagen–IL films.

Mechanical properties of films are largely associated with the distribution and density of intermolecular and intramolecular interactions in the network, so the effect of the ILs on the mechanical properties is shown in Table 4. It is found that the tensile strength decreases and elongation at break increases when [Ch][Seri] is added to collagen, in particular, for the films with the highest filler content, as shown in Figure 6.

**Table 4:** Tensile Strength (TS) and Elongation at Break (EB) of Collagen–IL Samples^a^

films	TS (MPa)	EB (%)
**control**	38.7 ± 2.0	20.8 ± 0.9
**10[Ch][Seri]**	24.4 ± 0.7	27.7 ± 1.1
**20[Ch][Seri]**	20.7 ± 0.7	36.0 ± 1.3
**40[Ch][Seri]**	3.2 ± 0.4	50.6 ± 1.5
**40[Ch][DHP]**	8.9 ± 0.5	45.9 ± 0.6
**40[Ch][TFSI]**	9.7 ± 0.6	26.7 ± 0.7
**40[EMIM][TFSI]**	13.1 ± 0.9	36.8 ± 0.7

a Two means followed by the same letter in the same column are not significantly (*P* > 0.05) different from Tukey’s multiple range test. *N* = 5 was the minimum number of replications.

These results are attributed to the presence of the IL, typically having a plasticizing effect on the polymer films and to the presence of water molecules, which enhance chain mobility, since the hygroscopic character of this IL increases the water holding capacity of the films.^74,75^ A similar effect to that of [Ch][Seri] can be observed in samples with [Ch][DHP], attributed to the role of ILs as plasticizers.^49^ Additionally, although a decrease in the tensile strength, TS, value is also shown in 40[Ch][TFSI] and 40[EMIM][TFSI] samples, the lower elongation-at-break, EB, values than those of 40[Ch][Seri] and 40[Ch][DHP] are attributed to the hydrophobic character of the [TFSI]^−^ anion and the migration of those ILs from the collagen matrix observed by WCA and SEM results. Also, for the same IL amount (40 wt %), the overall mechanical properties depend on the IL type. For the [Ch] cation, it is observed that the mechanical properties increase for IL with a low viscosity value due to the anion size. This behavior is also observed for the [EMIM] cation.

### Electrical Properties

**Figure 7:**
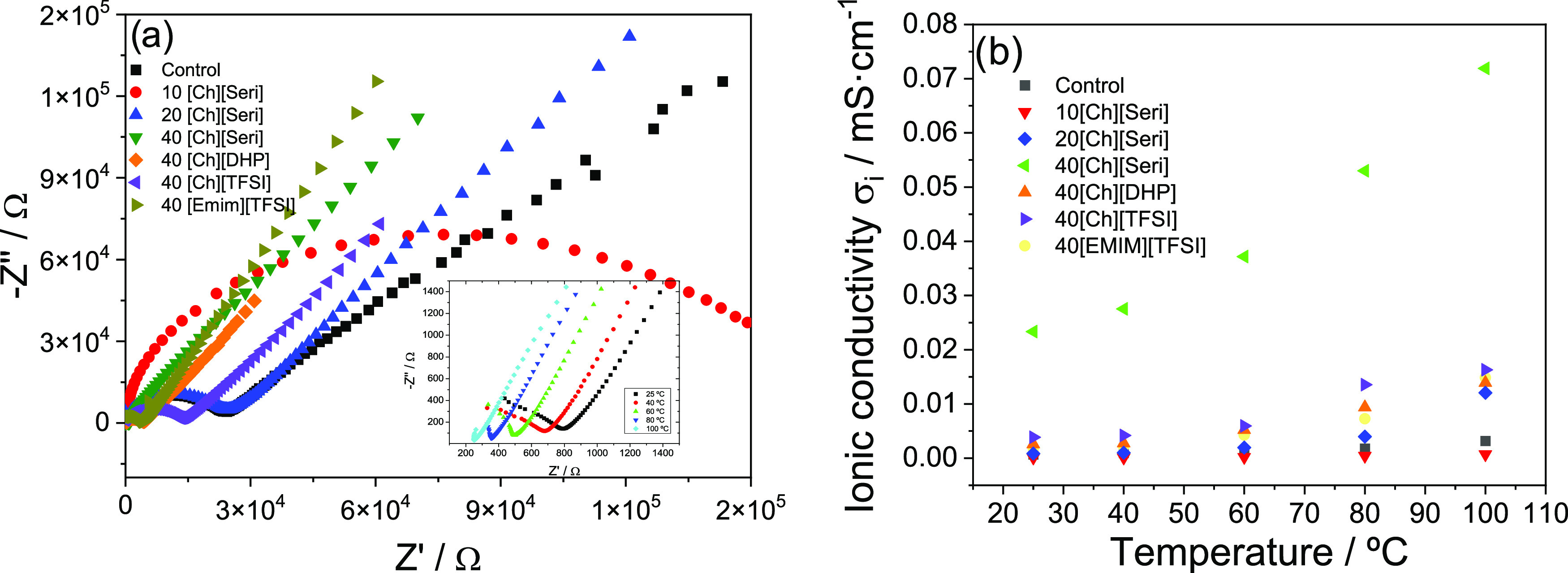
(a) Nyquist plot and (b) ionic conductivity value at different temperatures of collagen–IL films. The inset of (a) Nyquist plots for 40[Ch][Seri] as a function of temperature.

The electrical properties of the films were studied by impedance spectroscopy in the temperature range from 25 to 100 °C and the frequency range from 1 MHz to 10 mHz, the results being presented in the Nyquist plots in Figure 7a, which depend on the IL amount and type. The Nyquist plots allow us to identify processes with different time constants, the shape of the curves providing information about the possible electrical conduction mechanisms.

The Nyquist plots represented in Figure 7a show three characteristic parts defined by a semicircle located in the high-frequency range that corresponds to the charge-transfer process (bulk material properties), a transition controlled by the diffusion of counterions, and straight line for lower frequencies that is related to the diffusion process at the film/electrode interface.^76,77^ This behavior represented in Figure 7 depends on the IL content and type. For [Ch][Seri] IL, it is observed that the semicircle represented in Figure 7a decreases with increasing IL content, i.e., the intercepts on the *Z*′, real component, axis are dependent on the IL content. This decrease in the resistive part (ionic resistance calculated in the *Z*′ axis) is due to a decrease of the bulk resistance, i.e., an increase in the number of free charge carrier and their mobility. Furthermore, for the same IL content (40 wt %), it is observed that the semicircle depends on the IL type, which is related to the IL viscosity and ionic conductivity value. The inset of Figure 7a shows the Nyquist plot as a function of temperature for 40[Ch][Seri], the behavior being similar for the other films. It is observed that the semicircle decreases with increasing temperature due to the thermally activated increasing mobility of the ions, leading to a decrease in the electrical resistance.^78^

The ionic conductivity value as a function of temperature is shown in Figure 7b for the different collagen–IL films. It is observed that the ionic conductivity value increases with temperature due to a hopping conduction mechanism between local structural relaxations and segmental motions of the polymer–IL complexes. Further, increasing temperature leads to faster internal mode motions of the polymer chains, which also improves the electrical conductivity.^79^ At room temperature, the best ionic conductivity value of 0.023 mS cm^–1^ is obtained for the 40[Ch][Seri] sample. Furthermore, the ionic conductivity value depends on the anion size, as it affects its mobility correlated with van der Waals forces: for the same cation [Ch], the higher ionic conductivity value is observed for the anion with the smaller size.

### Touch Sensor Response

**Figure 8:**
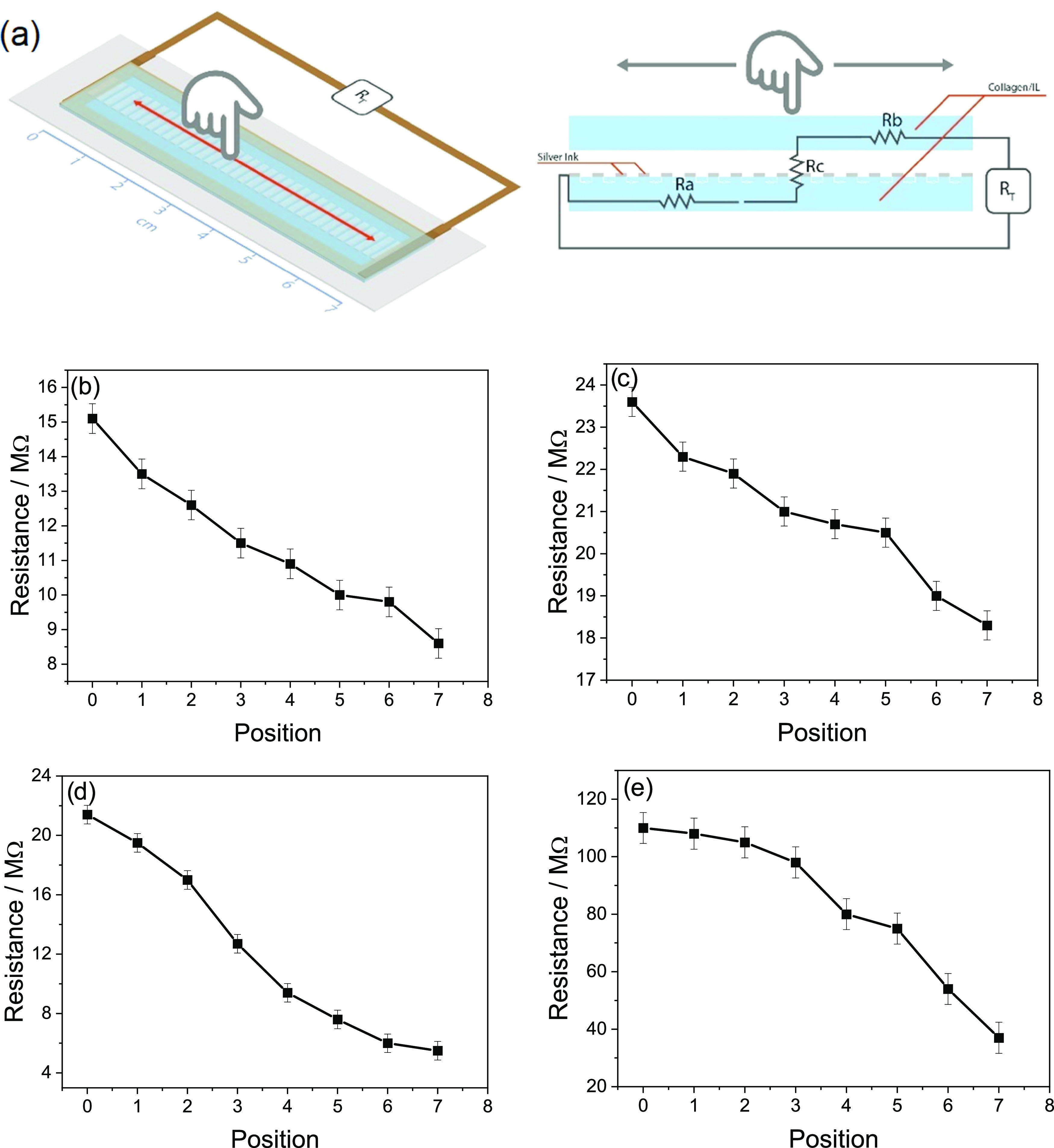
(a) Illustration of the working principle of the sensor based on the resistance variation and (b) resistance variation as a function of the position for (b) 40[Ch][Seri], (c) 40 [Ch][TFSI], (d) 40[Ch][DHP], and (e) 40[EMIM][TFSI].

Considering the electrical properties of the collagen–IL films, a digital multimeter Agilent 34401A was used to measure the resistance variation of the touch sensor device (Figure 1) in intervals of 10 mm with a constant applied pressure of 100 g, as shown in Figure 8a. These results demonstrate a suitable linearity between the resistance variation and the position for most of the samples, the response depending on the IL type, as can be seen in Figure 8b–e, as it is correlated with the ionic conductivity of the samples.

**Figure 9:**
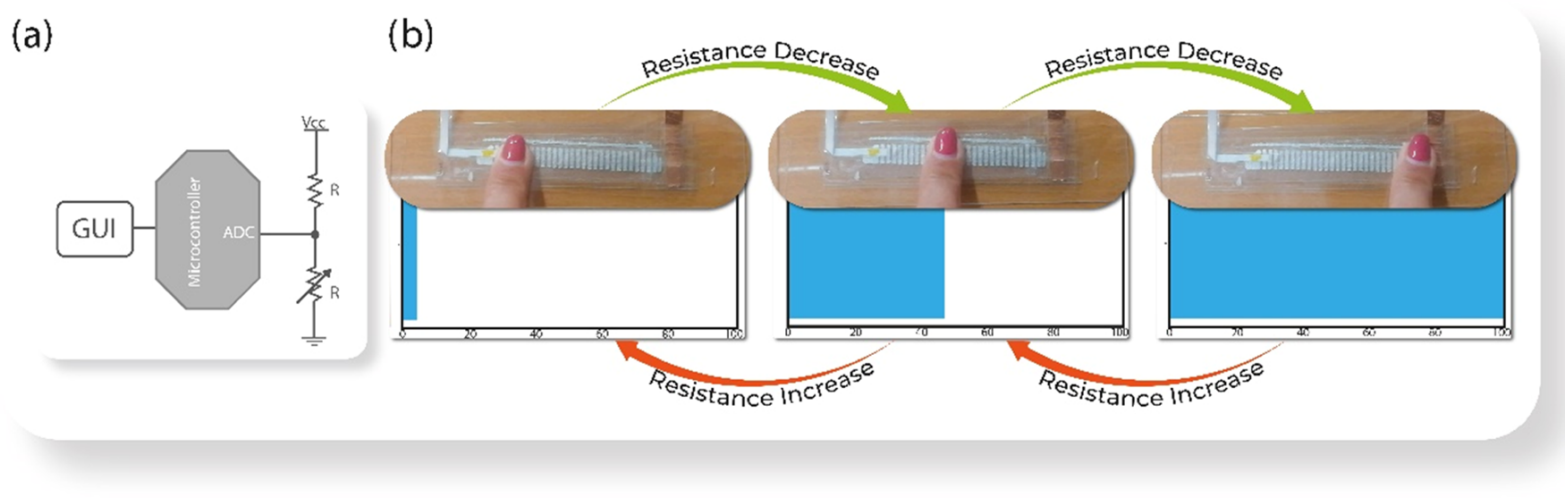
(a) Schematic representation of the electronic readout circuit. (b) Frames from the application of the sliding sensor, with the representation of 3% stages for the 40[Ch][Seri] sample.

The developed touch sensing device was connected to a microcontroller (Arduino UNO) through a voltage divider, allowing the measurement of the prototype as a sliding sensor or digital potentiometer (Figure 9a). The sliding sensor changes the resistance by pressing on the film, reducing the contact resistance between the top and bottom collagen–IL films (Figure 8a), the resistance variation being different for each position. Depending on the position of the finger (0–70 mm), the percentage of the bar varies from 0 to 100% in relation to the resistance variation (Figure 9b). A Python application was developed in order to show the sliding application in a progress bar. The microcontroller sends the received digital data from the analog-to-digital converter (ADC) through the universal serial bus to the computer, where the Python application receives the digital data. The data was converted to a percentage variation, as shown in Figure 9b for 40[Ch][Seri].

In order to maintain the last value when the finger is removed, the application only varies the bar chart percentage if the variation of the resistance is below a threshold. If it is not and the variation is too large, the algorithm assumes that the finger was removed from the slider and maintains the last position (see the Video on the Supporting Information).

Considering the actual interest in sustainability and environment-friendly materials, the proposed sensor exhibits lower environmental impact than recently reported pressure sensing materials^80^ since most of them are based on the combination of synthetic matrix, such as polyimide (PI),^81^ poly(dimethylsiloxane) (PDMS),^82^ perfluoroalkoxy alkane (PFA),^83^ poly(vinylidene difluoride) (PVDF),^84^ and fillers like carbon nanotubes (CNTs),^85,86^ metal nanowires (NWs),^87^ and metal nanoparticles (NPs).^88^ In terms of green chemistry, the primary goal of green techniques and technologies is to reduce the adverse effects of contaminants on the environment and living beings. In this context, although there are studies based on collagen for pressure sensing applications, the chemicals used in the processing of those composites are highly polluting to the environment.^89−91^ Specifically, Ma et al.^89^ prepared high-sensitivity microchannel-structured collagen fiber-based sensors where glutaraldehyde, Ag NPs, and HCl were used as a cross-linking agent, a filler, and a solvent, respectively. In this context, while it is true that some ionic liquids such as [EMIM][TFSI] and [Ch][TFSI] are toxic to some extent and nonbiodegradable, due to [EMIM]^+^, [TFSI]^−^ ions, the so-called bio-IL [Ch][DHP] and [Ch][Seri] were prepared using a green channel as sustainable, nontoxic, and biodegradable ILs.^92^

Aside from the potential benefits of environment-friendly products and processes, other challenges, such as the useful lifetime (life-cycle costs), impurities, impact of water content, thermal stability, aging, IL losses as well as recyclability data, are essential for the proper evaluation of the viability of ILs in commercial applications such as sustainable materials for multifunctional devices. Ionic liquids are expensive when compared to the mainly used components of electronics.^93^ Due to economic and environmental issues, the recovery and purification of ILs are essential.^94^ Moreover, the presence of impurities and water excess in some of the ionic liquids can lead to interference in the proper performance.^95^ The ILs used in this work are commercial, where [Ch][DHP] and [Ch][Seri] are technical grade and [Ch][TFSI] and [EMIM][TFSI] are purified grade. Although no effect of impurities on the characterization of the materials has been observed, the performance of the sensors should be further tested^96^ in real-operating conditions and in actual prototype device implementation.^95^ In this context, it is shown that IL–collagen films can be used as resistive touch sensors and that their electrical response can also be adjusted properly by varying the IL content and type, being suitable for the next generation of sustainable electronic devices.

## Conclusions

Sustainable collagen blends with different ionic liquids (ILs), sharing the same cation ([Ch][DHP], [Ch][TSI], [Ch][Seri]) and anion ([Emim][TFSI]), and IL contents from 0 to 40 wt % have been developed for resistive touch sensing applications. It is observed that different α-helix/β-sheet ratio values have been obtained for the different samples, indicating different physical interactions between the ILs and collagen matrix. This behavior is also confirmed by the FTIR and XRD analyses. The IL content and type do not affect the collagen triple helix structure that is responsible for the stability of collagen molecules. Regarding mechanical properties, ILs act as plasticizers for the collagen matrix. In addition, the IL content and type improve the ionic conductivity value, the best room-temperature ionic conductivity value of 0.023 mS cm^–1^ being obtained for the 40[Ch][Seri] sample. Considering the electrical response, the collagen–IL films have been implemented in a resistive touch sensing device, showing an excellent response, that can be tailored depending on the IL type. This work demonstrates that collagen–IL films are suitable for resistive touch sensing applications, being also suitable for the new generation of sustainable electronic materials based on biopolymers.

## Supporting Information Available

Application of the sliding sensor showing different percentage stages depending on the position of the finger (MP4)

## References

[ref1] Bhatnagar A.; Wagh S.; Singh B.; Agarwal R. R.; Khan F. Smart materials - a review. Ann. Dent. Spec. 2016, 4, 10–12.

[ref2] Bahl S.; Nagar H.; Singh I.; Sehgal S. In Smart Materials Types, Properties and Applications: A Review, International Conference on Aspects of Materials Science and Engineering (ICAMSE), Electr Network, May 29-30; Electr Network, 2020; pp 1302–1306.

[ref3] Maurya K. K.; Rawat A.; Jha G. In Smart Materials and Electro-Mechanical Impedance Technique: A Review, 2nd International Conference on Processing and Characterization of Materials (ICPCM), NIT Rourkela, Rourkela, INDIA; NIT Rourkela: Rourkela, INDIA, 2019; pp 4993–5000.

[ref4] Wang W.; Li P. F.; Xie R.; Ju X. J.; Liu Z.; Chu L. Y. Designable Micro-/Nano-Structured Smart Polymeric Materials. Adv. Mater. 2021, 34, 2107877 10.1002/adma.202107877. 34897843

[ref5] Correia D. M.; Fernandes L. C.; Fernandes M. M.; Hermenegildo B.; Meira R. M.; Ribeiro C.; Ribeiro S.; Reguera J.; Lanceros-Méndez S. Ionic Liquid-Based Materials for Biomedical Applications. Nanomaterials 2021, 11, 2401 10.3390/nano11092401. 34578716 PMC8471968

[ref6] Correia D. M.; Fernandes L. C.; Martins P. M.; García-Astrain C.; Costa C. M.; Reguera J.; Lanceros-Méndez S. Ionic Liquid–Polymer Composites: A New Platform for Multifunctional Applications. Adv. Funct. Mater. 2020, 30, 1909736 10.1002/adfm.201909736.

[ref7] Bhaskar Reddy A. V.; Moniruzzaman M.; Bustam M. A.; Goto M.; Saha B. B.; Janiak C. Ionic liquid polymer materials with tunable nanopores controlled by surfactant aggregates: a novel approach for CO2 capture. J. Mater. Chem. A 2020, 8, 15034–15041. 10.1039/C9TA13077B.

[ref8] Ejeromedoghene O.; Oderinde O.; Adewuyi S. Advances in polymeric ionic liquids-based smart polymeric materials: emerging fabrication strategies. Phys. Sci. Rev. 2022, 7, 759–772. 10.1515/psr-2020-0081.

[ref9] Pei Y. C.; Zhang Y. X.; Ma J.; Fan M. H.; Zhang S. J.; Wang J. J. Ionic liquids for advanced materials. Mater. Today Nano 2022, 17, 100159 10.1016/j.mtnano.2021.100159.

[ref10] Dong Y. Q.; Yeung K. W.; Tang C. Y.; Law W. C.; Tsui G. C. P.; Xie X. L. Development of ionic liquid-based electroactive polymer composites using nanotechnology. Nanotechnol. Rev. 2021, 10, 99–116. 10.1515/ntrev-2021-0009.

[ref11] Guerrero-Sanchez C.; Erdmenger T.; Lara-Ceniceros T.; Jimenez-Regalado E.; Schubert U. S. In Smart Materials Based on Ionic Liquids: The Magnetorheological Fluid Case, 236th National Meeting of the American-Chemical-Society, Philadelphia, PA, Aug 17-21; Philadelphia, PA, 2008; pp 147–155.

[ref12] Zhang S. G.; Zhang Q. H.; Zhang Y.; Chen Z. J.; Watanabe M.; Deng Y. Q. Beyond solvents and electrolytes: Ionic liquids-based advanced functional materials. Prog. Mater. Sci. 2016, 77, 80–124. 10.1016/j.pmatsci.2015.10.001.

[ref13] Cui J. C.; Li Y.; Chen D.; Zhan T. G.; Zhang K. D. Ionic Liquid-Based Stimuli-responsive Functional Materials. Adv. Funct. Mater. 2020, 30, 2005522 10.1002/adfm.202005522.

[ref14] Liu B. Y.; Jin N. X. The Applications of Ionic Liquid as Functional Material: A Review. Curr. Org. Chem. 2016, 20, 2109–2116. 10.2174/1385272820666160527101844.

[ref15] Correia D. M.; Lizundia E.; Meira R. M.; Rincón-Iglesias M.; Lanceros-Méndez S. Cellulose nanocrystal and water-soluble cellulose derivative based electromechanical bending actuators. Materials 2020, 13, 2294 10.3390/ma13102294. 32429292 PMC7287802

[ref16] Martins P.; Correia D. M.; Correia V.; Lanceros-Mendez S. Polymer-based actuators: back to the future. Phys. Chem. Chem. Phys. 2020, 22, 15163–15182. 10.1039/D0CP02436H. 32633288

[ref17] Correia D. M.; Serra R. S. I.; Tejedor J. A. G.; Bermudez V. D.; Balado A. A.; Meseguer-Duenas J. M.; Ribelles J. L. G.; Lanceros-Mendez S.; Costa C. M. Ionic and conformational mobility in poly(vinylidene fluoride)/ionic liquid blends: Dielectric and electrical conductivity behavior. Polymer 2018, 143, 164–172. 10.1016/j.polymer.2018.04.019.

[ref18] Keum K.; Heo J. S.; Eom J.; Lee K. W.; Park S. K.; Kim Y. H. Highly Sensitive Textile-Based Capacitive Pressure Sensors Using PVDF-HFP/Ionic Liquid Composite Films. Sensors 2021, 21, 442 10.3390/s21020442. 33435515 PMC7827140

[ref19] Meira R. M.; Correia D. M.; Ribeiro S.; Costa P.; Gomes A. C.; Gama F. M.; Lanceros-Mendez S.; Ribeiro C. Ionic-Liquid-Based Electroactive Polymer Composites for Muscle Tissue Engineering. ACS Appl. Polym. Mater. 2019, 1, 2649–2658. 10.1021/acsapm.9b00566.

[ref20] Sarkar R.; Kundu T. K. Density functional theory studies on PVDF/ionic liquid composite systems. J. Chem. Sci. 2018, 130, 115 10.1007/s12039-018-1522-4.

[ref21] Xu P.; Fu W. J.; Luo X.; Ding Y. S. Enhanced dc conductivity and conductivity relaxation in PVDF/ionic liquid composites. Mater. Lett. 2017, 206, 60–63. 10.1016/j.matlet.2017.06.104.

[ref22] Abd El-Messieh S. L.; Younan A. F.; Shafik E. S.; Rozik N. N. Ionic conductivity and mechanical Properties of ionic Liquids incorporated PMMA based Polymer Electrolytes. KGK, Kautsch. Gummi Kunstst. 2018, 71, 26–31.

[ref23] Puyu L. S. Z. Synthesis and application of ionic liquid as green plasticizer for PMMA. J. Polym. Mater. 2006, 23, 97–100.

[ref24] Scott M. P.; Rahman M.; Brazel C. S. Application of ionic liquids as low-volatility plasticizers for PMMA. Eur. Polym. J. 2003, 39, 1947–1953. 10.1016/S0014-3057(03)00129-0.

[ref25] Trigo-López M.; Vallejos S.; Ruiz J. A. R.; Ramos C.; Beltran S.; Garcia F. C.; Garcia J. M. Fabrication of microporous PMMA using ionic liquids: An improved route to classical ScCO2 foaming process. Polymer 2019, 183, 121867 10.1016/j.polymer.2019.121867.

[ref26] Kim Y. M.; Moon H. C. Ionoskins: nonvolatile, highly transparent, ultrastretchable ionic sensory platforms for wearable electronics. Adv. Funct. Mater. 2020, 30, 1907290 10.1002/adfm.201907290.

[ref27] Li S.; Liu H.; Zhu Z.; Sun X.; Tang Z.; Guo Y.; Hu Q.; Zhang Y. J. Voltage response of three ionic polymer pressure sensors based on ion migration at different ambient humidities. Smart Mater. Struct. 2020, 30, 025004 10.1088/1361-665X/abcca1.

[ref28] Fernandes L. C.; Correia D. M.; Pereira N.; Tubio C. R.; Lanceros-Méndez S. Highly sensitive transparent piezoionic materials and their applicability as printable pressure sensors. Compos. Sci. Technol. 2021, 214, 108976 10.1016/j.compscitech.2021.108976.

[ref29] Campos D. A.; Ribeiro T. B.; Teixeira J. A.; Pastrana L.; Pintado M. M. Integral Valorization of Pineapple (*Ananas comosus* L.) By-Products through a Green Chemistry Approach towards Added Value Ingredients. Foods 2020, 9, 60 10.3390/foods9010060. 31936041 PMC7022615

[ref30] Barbi S.; Macavei L. I.; Fuso A.; Luparelli A. V.; Caligiani A.; Ferrari A. M.; Maistrello L.; Montorsi M. Valorization of seasonal agri-food leftovers through insects. Sci. Total Environ. 2020, 709, 136209 10.1016/j.scitotenv.2019.136209. 31884276

[ref31] Communication from the Commission to the European Parliament, the Council, the European Economic and Social Committee, and the Committee of the Regions. A New Circular Economy Action Plan. For a Cleaner and More Competitive Europe; European Commission, 2020. https://eur-lex.europa.eu/resource.html?uri=cellar:9903b325-6388-11ea-b735-01aa75ed71a1.0017.02/DOC_1&format=PDF (accessed March 18, 2023).

[ref32] Mazur-Wierzbicka E. Circular economy: advancement of European Union countries. Environ. Sci. Eur. 2021, 33, 111 10.1186/s12302-021-00549-0.

[ref33] Wang H. A Review of the Effects of Collagen Treatment in Clinical Studies. Polymers 2021, 13, 3868 10.3390/polym13223868. 34833168 PMC8620403

[ref34] Brinckmann J.; Notbohm H.; Muller P. K. Collagen Suprastructures. In Collagen: Primer in Structure, Processing and Assembly; Springer, 2005; pp 185–205.

[ref35] Furtado M.; Chen L.; Chen Z.; Chen A.; Cui W. Development of fish collagen in tissue regeneration and drug delivery. Eng. Regener. 2022, 3, 217–231. 10.1016/j.engreg.2022.05.002.

[ref36] Tang C.; Zhou K.; Zhu Y.; Zhang W.; Xie Y.; Wang Z.; Zhou H.; Yang T.; Zhang Q.; Xu B. Collagen and its derivatives: From structure and properties to their applications in food industry. Food Hydrocolloids 2022, 131, 107748 10.1016/j.foodhyd.2022.107748.

[ref37] Henriksen K.; Karsdal M. Type I Collagen. In Biochemistry of Collagens, Laminins and Elastin, 2nd ed.; Academic Press, 2019; pp 1–11.

[ref38] Andonegi M.; Correia D. M.; Costa C. M.; Lanceros-Mendez S.; Caba K. D. L.; Guerrero P. Tailoring physicochemical properties of collagen-based composites with ionic liquids and wool for advanced applications. Polymer 2022, 252, 124943 10.1016/j.polymer.2022.124943.

[ref39] Guzzi Plepis A. M. D.; Goissis G.; Das-Gupta D. K. Dielectric and pyroelectric characterization of anionic and native collagen. Polym. Eng. Sci. 1996, 36, 2932–2938. 10.1002/pen.10694.

[ref40] de Campos Vidal B.; Mello M. L. S. Collagen type I amide I band infrared spectroscopy. Micron 2011, 42, 283–289. 10.1016/j.micron.2010.09.010. 21134761

[ref41] Duan R.; Zhang J.; Du X.; Yao X.; Konno K. Properties of collagen from skin, scale and bone of carp (Cyprinus carpio. Food Chem. 2009, 112, 702–706. 10.1016/j.foodchem.2008.06.020.

[ref42] Nagai T.; Saito M.; Tanoue Y.; Kai N.; Suzuki N. Characterization of Collagen from Sakhalin Taimen Skin as Useful Biomass. Food Technol. Biotechnol. 2020, 58, 445–454. 10.17113/ftb.58.04.20.6734. 33505207 PMC7821778

[ref43] Guerrero P.; Retegi A.; Gabilondo N.; de la Caba K. Mechanical and thermal properties of soy protein films processed by casting and compression. J. Food Eng. 2010, 100, 145–151. 10.1016/j.jfoodeng.2010.03.039.

[ref44] Rigual V.; Ovejero-Pérez A.; Rivas S.; Domínguez J. C.; Alonso M. V.; Oliet M.; Rodriguez F. Protic, Aprotic, and Choline-Derived Ionic Liquids: Toward Enhancing the Accessibility of Hardwood and Softwood. ACS Sustainable Chem. Eng. 2020, 8, 1362–1370. 10.1021/acssuschemeng.9b04443.

[ref45] Gautam R.; Kumar N.; Lynam J. G. Theoretical and experimental study of choline chloride-carboxylic acid deep eutectic solvents and their hydrogen bonds. J. Mol. Struct. 2020, 1222, 128849 10.1016/j.molstruc.2020.128849.

[ref46] Fujioka N.; Morimoto Y.; Arai T.; Kikuchi M. Discrimination between normal and malignant human gastric tissues by Fourier transform infrared spectroscopy. Cancer Detect. Prev. 2004, 28, 32–36. 10.1016/j.cdp.2003.11.004. 15041075

[ref47] Chakrapani V. Y.; Gnanamani A.; Giridev V. R.; Madhusoothanan M.; Sekaran G. Electrospinning of type I collagen and PCL nanofibers using acetic acid. J. Appl. Polym. Sci. 2012, 125, 3221–3227. 10.1002/app.36504.

[ref48] Mehta A.; Raghava Rao J.; Fathima N. N. Electrostatic Forces Mediated by Choline Dihydrogen Phosphate Stabilize Collagen. J. Phys. Chem. B 2015, 119, 12816–12827. 10.1021/acs.jpcb.5b07055. 26388068

[ref49] Reizabal A.; Correia D. M.; Costa C. M.; Perez-Alvarez L.; Vilas-Vilela J. L.; Lanceros-Méndez S. Silk Fibroin Bending Actuators as an Approach Toward Natural Polymer Based Active Materials. ACS Appl. Mater. Interfaces 2019, 11, 30197–30206. 10.1021/acsami.9b07533. 31330104

[ref50] Kamal Mohamed S. M.; Murali Sankar R.; Kiran M. S.; Jaisankar S. N.; Milow B.; Mandal A. B. Facile Preparation of Biocompatible and Transparent Silica Aerogels as Ionogels Using Choline Dihydrogen Phosphate Ionic Liquid. Appl. Sci. 2021, 11, 206 10.3390/app11010206.

[ref51] Onghena B.; Jacobs J.; Van Meervelt L.; Binnemans K. Homogeneous liquid–liquid extraction of neodymium(iii) by choline hexafluoroacetylacetonate in the ionic liquid choline bis(trifluoromethylsulfonyl)imide. Dalton Trans. 2014, 43, 11566–11578. 10.1039/C4DT01340A. 24938933

[ref52] Akai N.; Akio K.; Kazuhiko S. First Observation of the Matrix-isolated FTIR Spectrum of Vaporized Ionic Liquid: An Example of EmimTFSI, 1-Ethyl-3-methylimidazolium Bis(trifluoromethanesulfonyl)imide. Chem. Lett. 2008, 37, 256–257. 10.1246/cl.2008.256.

[ref53] Kiefer J.; Fries J.; Leipertz A. Experimental Vibrational Study of Imidazolium-Based Ionic Liquids: Raman and Infrared Spectra of 1-Ethyl-3-methylimidazolium Bis(Trifluoromethylsulfonyl)imide and 1-Ethyl-3-methylimidazolium Ethylsulfate. Appl. Spectrosc. 2007, 61, 1306–1311. 10.1366/000370207783292000. 18198022

[ref54] Cho B.-S.; Choi J.; Kim K.-Y. Preparation and properties of solid polymer electrolyte based on imidazolium-based ionic liquids for structural capacitors. Fibers Polym. 2017, 18, 1452–1458. 10.1007/s12221-017-7266-9.

[ref55] Sedlak P.; Sobola D.; Gajdos A.; Dallaev R.; Nebojsa A.; Kubersky P. Surface Analyses of PVDF/NMP/[EMIM][TFSI] Solid Polymer Electrolyte. Polymers 2021, 13, 2678 10.3390/polym13162678. 34451218 PMC8401855

[ref56] Tarannum A.; Rao J. R.; Fathima N. N. Choline-Based Amino Acid ILs–Collagen Interaction: Enunciating Its Role in Stabilization/Destabilization Phenomena. J. Phys. Chem. B 2018, 122, 1145–1151. 10.1021/acs.jpcb.7b10645. 29239608

[ref57] Tarannum A.; Adams A.; Blümich B.; Fathima N. N. Impact of Ionic Liquids on the Structure and Dynamics of Collagen. J. Phys. Chem. B 2018, 122, 1060–1065. 10.1021/acs.jpcb.7b09626. 29265818

[ref58] Fellows A. P.; Casford M. T. L.; Davies P. B. Spectral Analysis and Deconvolution of the Amide I Band of Proteins Presenting with High-Frequency Noise and Baseline Shifts. Appl. Spectrosc. 2020, 74, 597–615. 10.1177/0003702819898536. 31868519

[ref59] Gauza-Włodarczyk M.; Kubisz L.; Mielcarek S.; Włodarczyk D. Comparison of thermal properties of fish collagen and bovine collagen in the temperature range 298–670K. Mater. Sci. Eng.: C 2017, 80, 468–471. 10.1016/j.msec.2017.06.012. 28866189

[ref60] Schroepfer M.; Meyer M. DSC investigation of bovine hide collagen at varying degrees of crosslinking and humidities. Int. J. Biol. Macromol. 2017, 103, 120–128. 10.1016/j.ijbiomac.2017.04.124. 28499945

[ref61] Miles C. A.; Ghelashvili M. Polymer-in-a-Box Mechanism for the Thermal Stabilization of Collagen Molecules in Fibers. Biophys. J. 1999, 76, 3243–3252. 10.1016/S0006-3495(99)77476-X. 10354449 PMC1300293

[ref62] Miles C. A.; Avery N. C.; Rodin V. V.; Bailey A. J. The Increase in Denaturation Temperature Following Cross-linking of Collagen is Caused by Dehydration of the Fibres. J. Mol. Biol. 2005, 346, 551–556. 10.1016/j.jmb.2004.12.001. 15670603

[ref63] Shi D.; Liu F.; Yu Z.; Chang B.; Goff H. D.; Zhong F. Effect of aging treatment on the physicochemical properties of collagen films. Food Hydrocolloids 2019, 87, 436–447. 10.1016/j.foodhyd.2018.08.016.

[ref64] Ramachandran E.; Natarajan S. Crystal growth of some amino acids in gel: Crystallization of DL-serine and its characterization. Indian J. Pure Appl. Phys. 2005, 43, 372–376.

[ref65] Tarique J.; Sapuan S. M.; Khalina A. Effect of glycerol plasticizer loading on the physical, mechanical, thermal, and barrier properties of arrowroot (*Maranta arundinacea*) starch biopolymers. Sci. Rep. 2021, 11, 13900 10.1038/s41598-021-93094-y. 34230523 PMC8260728

[ref66] Tao D.-J.; Cheng Z.; Chen F.-F.; Li Z.-M.; Hu N.; Chen X.-S. Synthesis and Thermophysical Properties of Biocompatible Cholinium-Based Amino Acid Ionic Liquids. J. Chem. Eng. Data 2013, 58, 1542–1548. 10.1021/je301103d.

[ref67] Yoshizawa-Fujita M.; Fujita K.; Forsyth M.; MacFarlane D. R. A new class of proton-conducting ionic plastic crystals based on organic cations and dihydrogen phosphate. Electrochem. Commun. 2007, 9, 1202–1205. 10.1016/j.elecom.2007.01.024.

[ref68] Jeon J.-H.; Tanaka K.; Chujo Y. Synthesis of sulfonic acid-containing POSS and its filler effects for enhancing thermal stabilities and lowering melting temperatures of ionic liquids. J. Mater. Chem. A 2014, 2, 624–630. 10.1039/C3TA14039C.

[ref69] Meng Z.; Zheng X.; Tang K.; Liu J.; Ma Z.; Zhao Q. Dissolution and regeneration of collagen fibers using ionic liquid. Int. J. Biol. Macromol. 2012, 51, 440–448. 10.1016/j.ijbiomac.2012.05.030. 22676994

[ref70] Bak S. Y.; Lee S. W.; Choi C. H.; Kim H. W. Assessment of the Influence of Acetic Acid Residue on Type I Collagen during Isolation and Characterization. Materials 2018, 11, 2518 10.3390/ma11122518. 30545004 PMC6316942

[ref71] Zhang F.-L.; Wang A.; Li Z.; He S.; Shao L. Preparation and Characterisation of Collagen from Freshwater Fish Scales. Food Nutr. Sci. 2011, 02, 818–823. 10.4236/fns.2011.28112.

[ref72] Soares B. G.; Silva A. A.; Pereira J.; Livi S. Preparation of Epoxy/Jeffamine Networks Modified With Phosphonium Based Ionic Liquids. Macromol. Mater. Eng. 2015, 300, 312–319. 10.1002/mame.201400293.

[ref73] Hou L. X.; Wang S. Study on ionic liquid [bmim]PF6 and [hmim]PF6 as plasticizer for PVC paste resin. Polym. Bull. 2011, 67, 1273–1283. 10.1007/s00289-011-0490-3.

[ref74] Rahman M.; Brazel C. S. Ionic liquids: New generation stable plasticizers for poly(vinyl chloride. Polym. Degrad. Stab. 2006, 91, 3371–3382. 10.1016/j.polymdegradstab.2006.05.012.

[ref75] Veroutis E.; Merz S.; Eichel R. A.; Granwehr J. Intra- and inter-molecular interactions in choline-based ionic liquids studied by 1D and 2D NMR. J. Mol. Liq. 2021, 322, 114934 10.1016/j.molliq.2020.114934.

[ref76] Chang B.-Y.; Park S.-M. Electrochemical Impedance Spectroscopy. Ann. Rev. Anal. Chem. 2010, 3, 207–229. 10.1146/annurev.anchem.012809.102211. 20636040

[ref77] Park S.-M.; Yoo J.-S. Electrochemical Impedance Spectroscopy for better electrochemical measurements. Anal. Chem. 2003, 75, 445A–461A. 10.1021/ac0313973. 14619851

[ref78] Prager S.; Eyring H. Thermal Diffusion in Binary Systems. J. Chem. Phys. 1953, 21, 1347–1350. 10.1063/1.1699218.

[ref79] Silva M. M.; Barbosa P. C.; Rodrigues L. C.; Gonçalves A.; Costa C.; Fortunato E. Gelatin in electrochromic devices. Opt. Mater. 2010, 32, 719–722. 10.1016/j.optmat.2009.08.013.

[ref80] Andonegi M.; Irastorza A.; Izeta A.; Cabezudo S.; de la Caba K.; Guerrero P. A Green Approach towards Native Collagen Scaffolds: Environmental and Physicochemical Assessment. Polymers 2020, 12, 1597 10.3390/polym12071597. 32708371 PMC7408220

[ref81] Luo N.; Zhang J.; Ding X.; Zhou Z.; Zhang Q.; Zhang Y. T.; Chen S. C.; Hu J. L.; Zhao N. Textile-enabled highly reproducible flexible pressure sensors for cardiovascular monitoring. Adv. Mater. Technol. 2018, 3, 1700222 10.1002/admt.201700222.

[ref82] Hua Q.; Sun J.; Liu H.; Bao R.; Yu R.; Zhai J.; Pan C.; Wang Z. L. Skin-inspired highly stretchable and conformable matrix networks for multifunctional sensing. Nat. Commun. 2018, 9, 244 10.1038/s41467-017-02685-9. 29339793 PMC5770430

[ref83] Wu N.; Chen S.; Lin S.; Li W.; Xu Z.; Yuan F.; Huang L.; Hu B.; Zhou J. Theoretical study and structural optimization of a flexible piezoelectret-based pressure sensor. J. Mater. Chem. A 2018, 6, 5065–5070. 10.1039/C8TA00688A.

[ref84] Lee J. S.; Shin K.-Y.; Cheong O. J.; Kim J. H.; Jang J. Highly sensitive and multifunctional tactile sensor using free-standing ZnO/PVDF thin film with graphene electrodes for pressure and temperature monitoring. Sci. Rep. 2015, 5, 7887 10.1038/srep07887. 25601479 PMC4298719

[ref85] Yu S.; Wang X.; Xiang H.; Zhu L.; Tebyetekerwa M.; Zhu M. Superior piezoresistive strain sensing behaviors of carbon nanotubes in one-dimensional polymer fiber structure. Carbon 2018, 140, 1–9. 10.1016/j.carbon.2018.08.028.

[ref86] Jeong C.; Park Y.-B. Exfoliated graphite nanoplatelet-carbon nanotube hybrid composites for compression sensing. ACS Omega 2020, 5, 2630–2639. 10.1021/acsomega.9b03012. 32095686 PMC7033660

[ref87] Duan S.; Wang Z.; Zhang L.; Liu J.; Li C. A highly stretchable, sensitive, and transparent strain sensor based on binary hybrid network consisting of hierarchical multiscale metal nanowires. Adv. Mater. Technol. 2018, 3, 1800020 10.1002/admt.201800020.

[ref88] Zhao Y.; Yang Y.; Cui L.; Zheng F.; Song Q. Electroactive Au@ Ag nanoparticles driven electrochemical sensor for endogenous H2S detection. Biosens. Bioelectron. 2018, 117, 53–59. 10.1016/j.bios.2018.05.047. 29885580

[ref89] Ma J.; Pan Z.; Zhang W.; Fan Q.; Li W.; Liang H. High-Sensitivity Microchannel-Structured Collagen Fiber-Based Sensors with Antibacterial and Hydrophobic Properties. ACS Sustainable Chem. Eng. 2022, 10, 16814–16824. 10.1021/acssuschemeng.2c05292.

[ref90] Zhang W.; Pan Z.; Ma J.; Wei L.; Chen Z.; Wang J. Degradable Cross-Linked Collagen Fiber/MXene Composite Aerogels as a High-Performing Sensitive Pressure Sensor. ACS Sustainable Chem. Eng. 2022, 10, 1408–1418. 10.1021/acssuschemeng.1c05757.

[ref91] Ke L.; Wang Y.; Ye X.; Luo W.; Huang X.; Shi B. Collagen-based breathable, humidity-ultrastable and degradable on-skin device. J. Mater. Chem. C 2019, 7, 2548–2556. 10.1039/C8TC05630G.

[ref92] Mena I. F.; Diaz E.; Palomar J.; Rodriguez J. J.; Mohedano A. F. Cation and anion effect on the biodegradability and toxicity of imidazolium– and choline–based ionic liquids. Chemosphere 2020, 240, 124947 10.1016/j.chemosphere.2019.124947. 31568943

[ref93] Greer A. J.; Jacquemin J.; Hardacre C. Industrial Applications of Ionic Liquids. Molecules 2020, 25, 5207 10.3390/molecules25215207. 33182328 PMC7664896

[ref94] Samir I. A.-E. Ionic Liquids Recycling for Reuse. In Ionic Liquids; Scott T. H., Ed.; IntechOpen, 2011; pp 239–273.

[ref95] Wasilewski T.; Gębicki J.; Kamysz W. Prospects of ionic liquids application in electronic and bioelectronic nose instruments. TrAC, Trends Anal. Chem. 2017, 93, 23–36. 10.1016/j.trac.2017.05.010.

[ref96] Singh S. K.; Savoy A. W. Ionic liquids synthesis and applications: An overview. J. Mol. Liq. 2020, 297, 112038 10.1016/j.molliq.2019.112038.

